# Magnetic resonance imaging in the prediction of aggressive histological features in papillary thyroid carcinoma

**DOI:** 10.1097/MD.0000000000011279

**Published:** 2018-06-29

**Authors:** Bin Song, Hao Wang, Yongqi Chen, Weiyan Liu, Ran Wei, Zedong Dai, Wenjuan Hu, Yi Ding, Lanyun Wang

**Affiliations:** a Department of Radiology; b Department of Pathology; c Department of General Surgery, Minhang Branch, Zhongshan Hospital, Fudan University, Shanghai, China.

**Keywords:** diagnosis, magnetic resonance imaging, pathology, thyroid cancer, thyroid nodule

## Abstract

To identify magnetic resonance imaging (MRI) features in the prediction of tumor aggressiveness in patients with papillary thyroid carcinoma (PTC).

In this prospective study, 105 patients with 122 PTCs underwent MRI with T1-weighted, T2-weighted, diffusion-weighted imaging and contrast-enhanced sequences prior to thyroidectomy. Based on exclusion criteria, 62 patients with 62 PTCs were finally suitable for further analysis. Tumor aggressiveness was defined according to the surgical histopathology. Tumor size, apparent diffusion coefficients (ADC) value and MRI features on images were obtained for each patient. Descriptive statistics for tumor aggressiveness, sensitivity, specificity, and accuracy of individual features were determined. A multivariate logistic regression model was developed to identify features that were independently predictive for tumor aggressiveness. Analyses of receiver-operating characteristic (ROC) curve were performed.

High aggressive PTC significantly differed from low aggressive PTC in size (*P* = .016), size classification (*P* < .001), ADC value (*P* = .01), angulation on the lateral surface of the lesion (*P* = .009), signal intensity heterogeneity on ADC maps (*P* = .003), early enhancement degree (*P* < .001), tumor margin on delayed contrast-enhanced images (*P* < .001), and inner lining of delayed ring enhancement (*P* = .028). The interobserver agreement between the 2 readers was satisfactory with Cohen k ranging from 0.83 to 1.00 (*P* < .001). Logistic regression model showed lesion size classification and tumor margin on delayed contrast-enhanced images as strongest independent predictors of high aggressive PTC (*P* = .009 and *P* = .047), with an accuracy of 83.9%. The area under ROC curve for ADC value and lesion size were 0.68 and 0.81, respectively.

These findings suggest that MRI before surgery has the potential to discriminate tumor aggressiveness in patients with PTC.

## 1 Introduction

Papillary thyroid carcinoma (PTC) is the most common form of thyroid cancer, ranging 65% to 88% in the United States and 87.8% to 92.8% in Eastern China.^[1,2]^ PTC has a favorable prognosis, with a mortality of 1% to 2% at 20 years.^[3]^ Patients with low aggressive PTC have a disease-specific survival rate of more than 99%.^[4]^ Surgery is the most important treatment available that influences the prognosis of PTC.^[5]^ Thyroidectomy without prophylactic central neck dissection (PCND) may be appropriate for small (T1 or T2), noninvasive, clinically node-negative (cN0) PTC. Thyroid lobectomy alone may be sufficient for the initial treatment of low aggressive PTC.^[6]^ According to a report,^[7]^ watchful-waiting approaches should be considered a research priority, which is a preferable option for patients with low aggressive PTCs. Therefore, initial therapeutic decision-making for PTC depends on the pre-operative risk stratification and is designed to evaluate the presence of PTC with aggressive features such as extrathyroidal extension (ETE), regional metastases, and distant metastases. Hence, accurate risk stratification and predicting tumor aggressiveness is the cornerstone for decision making in the management of thyroid cancer.^[6]^ This subsequently helps us to decide accurate therapeutic action for PTC.

Fine-needle aspiration (FNA) is the one of the most accurate and cost-effective method for evaluating thyroid nodules, but this provides only minimal information with regard to tumor aggressiveness.^[8]^ Ultrasound (US) is used as a diagnostic tool of thyroid nodules.^[9,10]^ However, US evaluation is an operator-dependent procedure and cannot always be relied upon as images of deep anatomic structures and those that are acoustically shadowed by bone or air cannot be produced.^[6]^ Additionally, routine neck ultrasonography cannot reliably exclude minor ETE.^[11,12]^ Computed tomography (CT) makes its use limited in the preoperative evaluation of thyroid nodules due to ionizing radiation and use of iodinated contrast agent. Advantages of magnetic resonance imaging (MRI) over CT are multiplane evaluation, better tissue contrast, and no radiation to the thyroid gland. Hence, MRI can be considered as a useful diagnostic tool for PTC.^[9]^

The relationship between MRI features and pathologic findings has been studied in specific types of tumors.^[13,14]^ However, the correlation of MRI features and aggressive behavior of PTC has been assessed to a limited extent. According to a few reports, apparent diffusion coefficient (ADC) values derived from diffusion-weighted magnetic resonance imaging (DWI) are related to tumor aggressive behavior.^[15]^ Our study advances prior work by comprehensive assessment of the accuracy of available MRI features of PTC (on non-enhanced and contrast-enhanced images) for prediction of high aggressive PTC. Our current study hypothesized the use of MRI in the prediction of high aggressive behavior of PTC. Hence, the purpose of this study was to explore whether the MRI features before surgery can be used to stratify high aggressive PTC from low aggressive PTC.

## 2 Methods

### 2.1 Study population

From January 2014 to January 2016, 111 consecutive patients undergoing surgical consultation for thyroidectomy on the basis of thyroid nodule FNA or US examination demonstrating either PTC or suspicious for thyroid cancer in our institution were offered enrollment in a prospective clinical study. This prospective protocol was approved by the Institutional Review Board of Minhang Branch, Zhongshan Hospital, Fudan University. After providing appropriate informed consent, 105 patients underwent enhanced MRI prior to thyroid surgery. There were 6 patients who had not undergone enhanced MRI prior to thyroid surgery, including 1 patient with claustrophobia, 3 patients with presence of contraindication to MRI, and 2 patients who refused to undergo MRI. Thyroidectomy was subsequently performed at our institution within 1 week of the MRI scan. The exclusion criteria were: lesions size less than 7 mm, which cannot be observed on MRI image; lesions with poor image quality deemed to be non-diagnostic after reviewing the images; smaller lesions in patients with multifocal PTCs due to overestimation of aggressiveness; and non PTC confirmed by pathology.

### 2.2 Histopathologic analysis

Surgical specimens of PTC after thyroidectomy with PCND were collected by a pathologist (YQC) who had more than 10 years of experience in PTC analysis based on the pathologic findings. Paraffin-embedded tissue blocks were obtained for each surgically resected specimen by sectioning each tumor. The section of each PTC was stained with hematoxylin and eosin (H&E). The pathologist reviewed the H&E sections of each PTC and evaluated tumor aggressiveness. Based on the ATA 2015 risk stratification system for differentiating thyroid carcinoma,^[6]^ tumor aggressiveness was evaluated individually using the following histopathologic features: aggressive histology (e.g., tall cell, hobnail variant, columnar cell carcinoma), vascular and/or tumor capsular invasion, ETE, regional metastases, and distant metastases. In our study, low aggressive group considered PTCs without histopathologic features of tumor aggressiveness, and high aggressive group considered the presence of any one of the histopathologic features of tumor aggressiveness. In order to establish the presence or absence of distant metastases, we followed up the patients for at least one year who received neck ultrasonography, blood thyroglobulin (Tg), and thyroglobulin antibody (TgAb) tests.

### 2.3 MRI protocol

MRI examination was performed with a GE EXCITE HD 1.5 T MR scanner (GE healthcare, Milwaukee, WI) using an eight-channel special neck coil (Chenguang Medical Technology Ltd, Shanghai, China). Routine MRI was performed with the following parameters: coronal T2-weighted fast recovery fast spin-echo (FRFSE) with fat suppression (TR = 1,280 ms, TE = 85 ms, thickness = 4 mm, spacing = 1 mm, matrix = 288 × 192, NEX = 4, FOV = 22 cm), and axial T1-weighted fast spin-echo (FSE) (TR = 460 ms, TE = 8 ms, thickness = 4 mm, spacing = 0.5 mm, NEX = 2, FOV = 25 cm, matrix = 288 × 192); and axial T2-weighted FRFSE with fat suppression (TR = 3000 ms, TE = 85 ms, thickness = 4 mm, spacing = 0.5 mm, NEX = 4, FOV = 25 cm, matrix = 320 × 224). DWI was performed using single-shot spin echo, echo planar imaging (EPI) sequence with the following diffusion gradient b factors: 800 s/mm^2^. Imaging parameters for DWI were the following: TR = 6,550 ms; TE = minimum; FOV = 25 cm; NEX = 6; matrix = 128 × 128; slice thickness = 4 mm; spacing = 0.5; diffusion direction = all; and examination time of DWI = 2.44 minutes. Contrast enhancement studies were performed using axial T1-weighted images which were obtained with a fast-spoiled gradient recalled echo (FSPGR) (TR = 5.7 ms, TE = 1.7 ms, FOV = 25 cm, matrix = 192 × 256, NEX = 1). Gadolinium (Magnevist, Bayer HealthCare, Berlin, Germany) was injected intravenously at a dose of 0.2 mL/kg body weight, and at an injection rate of 3 mL/s, followed by a 20-mL saline flush. In each patient, a single scan was performed prior to the injection of contrast medium. Six scans were obtained after injection of contrast at 30, 60,120,180, 240, and 300 seconds, respectively. In the contrast-enhanced protocol, holding breath was performed during each scanning phase.

### 2.4 Image analysis

All images were reviewed by 2 experienced radiologists (WJH and HW) with 8 and 13 years of experience in MRI reports, who were blinded to the pathologic results and recorded the MRI features of each PTC. If disagreements emerged, the 2 radiologists consulted with each other to reach a consensus. First, the images were obtained with each MRI sequence (T1-weighted, T2-weighted, DWI, and contrast enhanced T1-weighted) and recorded as nondiagnostic (with artifacts) and diagnostic (with no substantial artifacts). Second, age, sex, lesion location (right lobe, left lobe, isthmus), ADC value of lesion, and lesion size (greatest linear dimension of tumor, categorized as, ≤1 cm or > 1 cm) were recorded. Next, the following features were observed on the non-enhanced images (T1-weighted, T2-weighted, DWI and ADC): signal intensity heterogeneity (homogeneous, heterogeneous) and lesion shape (roundlike, irregular). The presence or absence of angulation on the lateral surface of lesion (defined as the appearance of an angulation on the local lateral surface of the lesion beyond the thyroid contour), fat plane between lesion and adjacent structures, were recorded. Finally, on contrast-enhanced images, tumor margin on delayed contrast-enhanced images, early enhancement degree, delayed enhancement pattern, and inner lining of the delayed ring enhancement were evaluated. The sequence obtained at 30 seconds after contrast agent injection was observed as early enhanced image, while the sequence obtained at 300 seconds was observed as delayed enhanced image. The tumor margin was classified as partly or poorly defined, well defined. By comparing with the normal thyroid tissue, early enhancement degree was classified as hyperenhanced, isoenhanced, and hypoenhanced. Delayed enhancement pattern was classified as delayed ring enhancement or delayed uniform enhancement. A delayed ring enhancement was defined as a ring-like pattern with a stronger enhancement in the peripheral portion of the mass than the central portion. A delayed uniform enhancement was defined as a pattern with homogeneous enhancement in most of the masses. The inner lining of the delayed ring enhancement was classified as smooth or indistinct.

ADC values were calculated by placing regions of interest (ROIs) within the thyroid lesions. Adjustments of the window level and width, as well as the magnification, were allowed. Circular ROIs within the most solid tumor part in the largest slice of the lesion on ADC maps were drawn. The solid tumor part showed a high signal intensity identified on DWI images. The ROIs were placed on PTC avoiding obvious cystic changes, hemorrhagic, areas of calcification, and lesion margins. The size of ROIs was determined to correspond with the blackest portion of lesions on ADC maps. The ROIs ranged in size from 10 to 100 mm^2^. The ADC values were measured twice and then the average value was taken.

### 2.5 Statistical analysis

First, descriptive statistical analyses were performed to analyze the data. Data with normal distribution were presented as mean ± standard deviation (SD); non-normal variables were reported as median (range). Student *t* test was used to compare the continuous variables of the 2 groups. A nonparametric Mann–Whitney *U* test was used to analyze the ordered multi categorical variables. Chi-square tests and Fisher exact test were used to compare the categorical variables of MRI features. Second, the sensitivity, specificity and accuracy of MRI features in the differentiation of high and low aggressive groups were determined. Interobserver agreement between the 2 readers was evaluated according to the Cohen k coefficient. A k statistic of 0.8 to 1 was considered indicative of excellent agreement; 0.6 to 0.79, good agreement; 0.4 to 0.59, moderate agreement; 0.2 to 0.39, fair agreement; and 0 to 0.19, poor agreement.^[16]^ Finally, multivariate logistic regression modeling was used to incorporate available imaging features to identify parameters that were independently predictive of high aggressive PTC. The variables that demonstrated a significant association with high aggressive status entered into the forward stepwise method. The final model was selected on the basis of the variables with *P* values < .05 or the ones that improved the model according to the likelihood ratio. The odds ratio was used as a measure of the relative magnitude of an association between predictor variables and high aggressive status. Receiver operator characteristic (ROC) curves were obtained at a cutoff value for differentiating high aggressive from low aggressive PTC.

A statistical analysis was performed using statistical software packages (IBM SPSS Statistics version 23). *P* values < .05 were considered statistically significant.

## 3 Results

**Figure 1:**
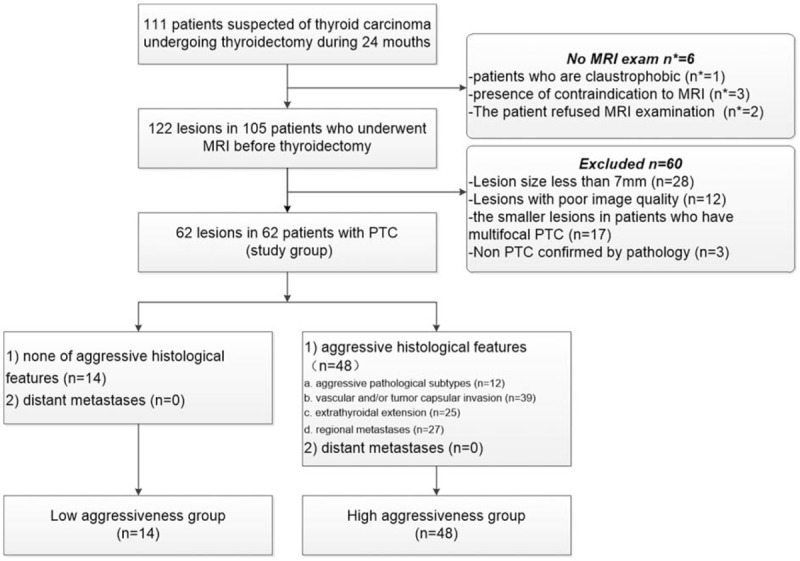
Flowchart outlines study group selection. MRI = magnetic resonance imaging. PTC = papillary thyroid carcinoma. n^∗^ = the number of patients. n = the number of lesions.

Of the 105 patients enrolled in the study, 122 lesions were included. Of these, 60 lesions were excluded due to the exclusion criteria, and a total of 62 patients with 62 lesions were included in our study (5 hobnail variant PTC, 1 columnar cell variant PTC, and 6 with the tall cell features/variant, 39 lesions with vascular and/or tumor capsular invasion, 25 lesions with ETE, 27 lesions with local lymph node metastasis) (Fig. 1). Overall mean age of the patients was 43.1 years, with an age range of 15 to 71 years. No significant differences were observed in the age of female (N = 44) and male (N = 18) patients (*P* = .085).

**Figure 2:**
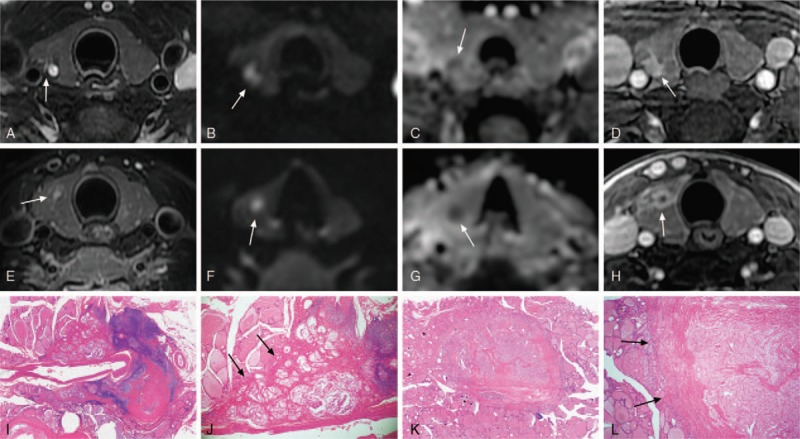
Images in a 28-year-old man (A–D, I,J) and a 37-year-old-woman (E–H,K,L) with low aggressive papillary thyroid carcinoma (PTC). The MR images show typical features of a low aggressive PTC, including well-defined margin on delayed contrast-enhanced images, homogeneous signal intensity on apparent diffusion coefficients (ADC) maps, and size less than 1 cm (8 mm, 8.3 mm). A, Axial T2-weighted MR image shows an irregular mass with indistinct margins and heterogeneous signal intensity (arrow). B, Axial diffusion-weighted imaging (DWI) image shows high homogeneous signal intensity (arrow). C, ADC map shows homogeneous signal intensity with the ADC value 1.39 × 10^−3^mm^2^/s (arrow). D, Axial delayed contrast-enhanced T1-weighted image shows uniform enhancement with well-defined margins (arrow). E, Axial T2-weighted MR image shows a round mass with indistinct margins and heterogeneous signal intensity (arrow). F, Axial DWI image shows high homogeneous signal intensity (arrow). G, ADC map shows homogeneous signal intensity with the ADC value 1.11 × 10^−3^mm^2^/s (arrow). H, Axial delayed contrast-enhanced T1-weighted image shows ring enhancement with well-defined margin and smooth inner lining (arrow). I,J, In this microscopic image (hematoxylin-eosin stain; original magnification, ×1 and ×40), the tumor with irregular shape consists of tumor cells and fibrous stroma. The fibrous stroma around the tumor is relatively thick and intact, and it is clear to the adjacent normal thyroid tissue (arrow). K,L, In this microscopic image (hematoxylin-eosin stain; original magnification, ×1 and ×40), the fibrous stroma surrounding the lesion is more than the center. The boundary between tumor and adjacent normal thyroid tissue is clearly visible (arrow), possibly accounting for the well-defined margin on delayed contrast-enhanced images.

**Figure 3:**
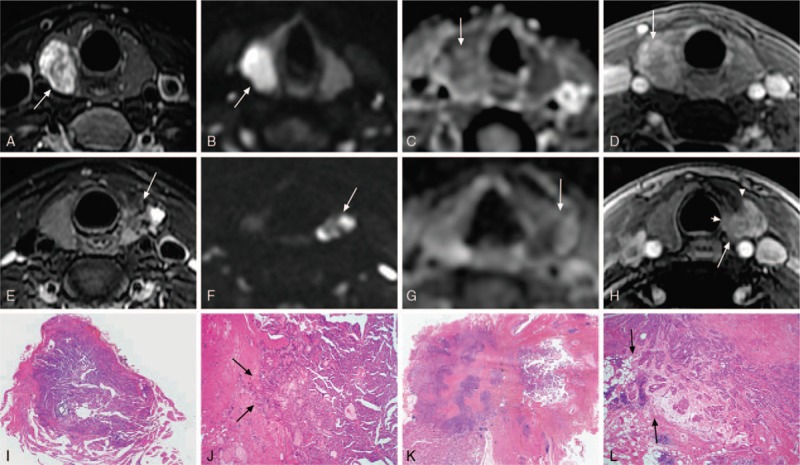
Images in a 29-year-old woman (A–D,I,J) and a 52-year-old-woman (E–H,K,L) with high aggressive papillary thyroid carcinoma (PTC). The MR images show typical features of a high aggressive PTC, including partly or poorly defined margin on delayed contrast-enhanced images, heterogeneous signal intensity on apparent diffusion coefficients (ADC) maps, and size larger than 1 cm (1.8 cm, 1.9 cm). A, Axial T2-weighted MR image shows a round-like mass with smooth margins and heterogeneous signal intensity (arrow). B, Axial diffusion-weighted imaging (DWI) image shows homogeneous high signal intensity (arrow). C, ADC map shows heterogeneous signal intensity with the minimum ADC value 1.38 × 10^−3^mm^2^/s (arrow). D, Axial delayed contrast-enhanced T1-weighted image shows ring enhancement with partly or poorly defined margins and indistinct inner lining (arrow). E, Axial T2-weighted MR image shows an irregular mass with indistinct margins and heterogeneous signal intensity (arrow). F, Axial DWI image shows high heterogeneous signal intensity (arrow). G, ADC map shows heterogeneous signal intensity with the minimum ADC value 0.79 × 10^−3^mm^2^/s (arrow). H, Axial delayed contrast-enhanced T1-weighted image shows mass enhancement with partly or poorly defined margin (long arrow), angulation on the lateral surface of the lesion (arrowhead) and absence of fat plane between lesion and trachea (short arrow). I,J, In this microscopic image (hematoxylin-eosin stain; original magnification, × 1 and × 40), the tumor consists of dense tumor cells and a little fibrous stroma. The malignant cells are seen infiltrating adjacent normal thyroid tissue (arrow). K,L, In this microscopic image (hematoxylin-eosin stain; original magnification, × 1 and × 40), the fibrous stroma in the center of lesion is more than the peripheral of the lesion. The malignant cells are seen infiltrating fat tissue (arrow). The boundary between tumor and adjacent normal thyroid tissue is indistinct, possibly accounting for the partly or poorly defined margin on delayed contrast-enhanced images.

Table 1 summarized the baseline characteristics and MRI features observed in the lesions with low or high aggressive PTCs. No significant differences were found between low and high aggressive PTCs with respect to sex (*P* = .295), age (*P* = .424), location (*P* = .604), shape (*P* = .092), fat plane between lesion and adjacent structures (*P* = .186), signal intensity heterogeneity in T1-weighted images, *P* = .531 and T2-weighted images, *P* = .538 and DWI images, *P* = .058, respectively, and delayed enhancement pattern (*P* = .258). The size of the lesions was bigger than 1 cm and significantly higher in the high aggressive PTC (75%) group than in the low aggressive PTC (14%; *P* < .001). High aggressive PTC differed significantly from low aggressive PTC in the angulation on the lateral surface of the lesion in all 20 lesions with angulation on the lateral surface were highly aggressive, *P* = .009, heterogeneous signal intensity on ADC maps was more common in high aggressive PTC, 96%, *P* = .003, early enhancement degree in all 5 lesions with hypoenhancement on early contrast-enhanced images were highly aggressive, and all 5 lesions with hyperenhancement were low aggressive, *P* < .001, tumor margin on delayed contrast-enhanced images in high aggressive PTC was more likely to have partly or poorly defined margin, *P* < .001, the inner lining of the delayed ring enhancement in high aggressive PTC exhibited indistinct inner lining more frequently, *P* = .028 (Figs. 2 and 3).

**Table 1:**
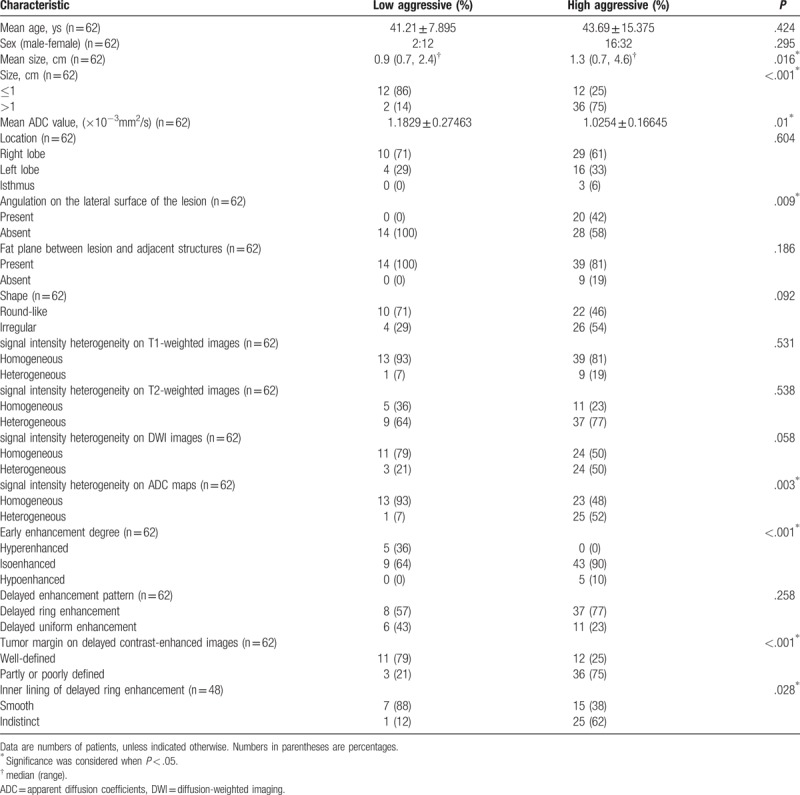
Tumor Characteristics in the Low and High aggressive groups.

Table 2 displayed the sensitivity, specificity, accuracy, and Cohen k coefficient of each individual with significant MRI features for differentiating high and low aggressive PTCs. Lesions bigger than 1 cm and a poorly or partly defined tumor margin on delayed contrast-enhanced images offered the highest combination of sensitivity, specificity and accuracy for characterization of PTC as high aggressiveness (75%, 85.7%, and 77.5%; 75%, 78.6%, and 75.8%; respectively). Although rarely present, angulation on the lateral surface of lesion and hypoenhancement on early contrast-enhanced images were specific features for characterization of PTC due to high aggressiveness. The interobserver agreement between the 2 readers was satisfactory with Cohen k ranging from 0.83 to 1.00 (*P* < .001 for the 6 MRI features).

**Table 2:**
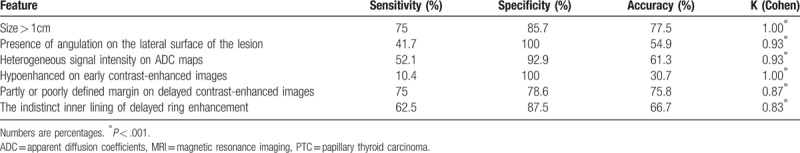
Sensitivity, specificity, accuracy and Cohen k coefficient of statistically significant MRI Feature for Prediction of high aggressive PTC.

Table 3 showed the results of the final logistic regression model. Of the factors studied in the logistic regression model, lesion size classification and tumor margin on delayed contrast-enhanced images were the strongest independent indicators of highly aggressive PTC (odds ratio [OR]: 10; 95% confidence interval [CI]: 1.776, 56.316; *P* = .009 and OR: 5; 95% CI: 1.023, 24.436; *P* = .047), with an accuracy of 83.9%.

**Table 3:**
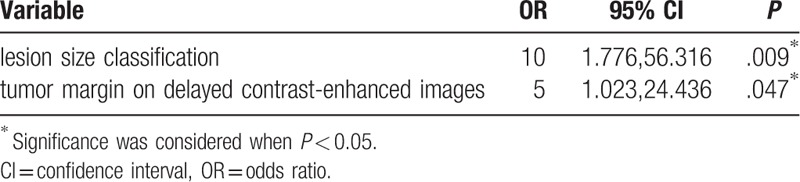
Results of final multivariate regression model.

**Figure 4:**
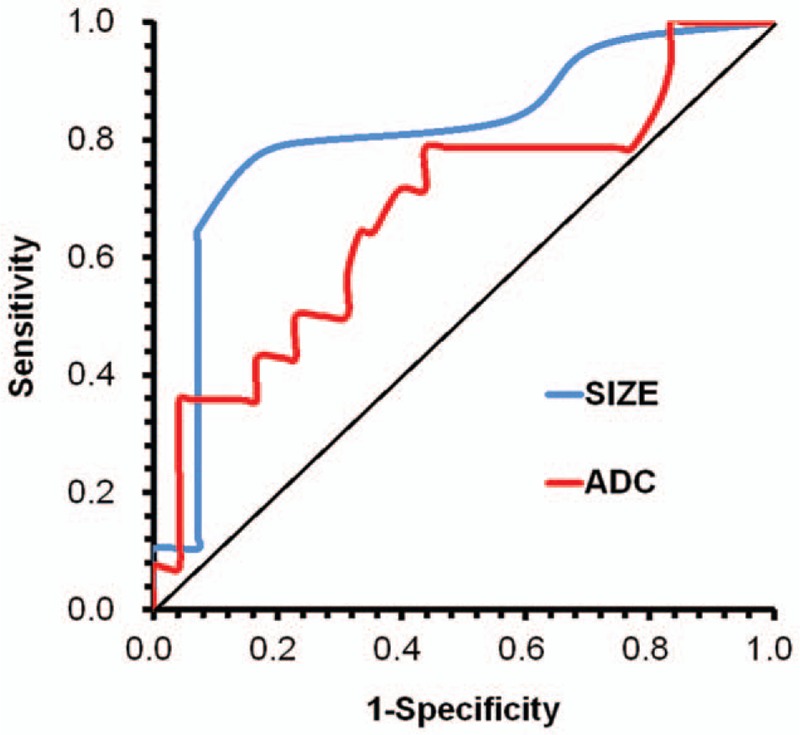
ROC curve of the ADC value (b-800) and tumor size used for differentiating high aggressive from low aggressive PTC. The AUC of the ADC value and tumor size were 0.68 and 0.81, respectively. ROC = Receiver operator characteristic. ADC = apparent diffusion coefficients. PTC = papillary thyroid carcinoma. AUC = area under the curve.

The mean ADC ± SD of low and high aggressive PTCs were 1.18 ± 0.27 × 10^−3^ mm^2^/s (range, 0.85–1.91 × 10^−3^ mm^2^/s) and 1.03 ± 0.17 × 10^−3^ mm^2^/s (range, 0.69–1.43 × 10^−3^ mm^2^/s), respectively. There was a significant difference observed between low and high aggressive PTCs (*P* = .01). The area under the curve (AUC) for ADC was 0.68 (Fig. 4). Based on the ROC curve, the best cut-off value was determined as 1.075 × 10^−3^ mm^2^/s for differentiating low and high aggressive PTCs. With ADCs less than 1.075 × 10^−3^ mm^2^/s, the sensitivity and specificity for diagnosing high aggressive PTC were 71.4% and 60.4%, respectively. The mean size ± SD of low and high aggressive PTCs were 0.96 ± 0.43 cm (range, 0.7–1.1 cm) and 1.45 ± 0.7 cm (range, 0.7–4.6 cm), respectively. There was significant difference observed between low and high aggressive PTCs (*P* = .016). The AUC for tumor size was 0.81 (Fig. 4). Based on the ROC curve, the best cut-off value of 1.05 cm was determined as for differentiating low and high aggressive PTCs. With size larger than 1.05 cm, the sensitivity and specificity for diagnosing high aggressive PTC were 75% and 85.7%, respectively.

## 4 Discussion

For PTC patients, grading of tumor aggressiveness is critical information needed for treatment planning, as it is correlated with the extent of surgery (lobectomy vs near total or total thyroidectomy) and whether or not PCND is needed for removal of lymph nodes needs to be studied. The risks of near total or total thyroidectomy are significantly greater than that of thyroid lobectomy.^[17]^ The most salient argument against routine application of PCND is that it may increase the risk of complications of thyroidectomy, such as recurrent laryngeal nerve injury and hypoparathyroidism, especially if the surgeon is inexperienced in the procedure.^[18]^ Thyroid lobectomy without PCND may be sufficient as initial treatment for low aggressive PTC. The results of our study demonstrated that a prediction model derived for lesion size classification and tumor margin on delayed contrast-enhanced images demonstrated as a useful tool for estimating the probability of high or low aggressive PTC, which allowed the clinician to identify patients that are likely to benefit from more aggressive initial therapy.

Prior investigators have identified the differentiating MRI features for low and high aggressive PTCs.^[15]^ Our study confirmed the importance of tumor margin and size in predicting the aggressiveness. Generally, a poorly or partly defined tumor margin on delayed contrast-enhanced images indicate that the fibrous stroma around the tumor was not complete and tumor cells have been infiltrated into the surrounding normal thyroid tissues, which shows high aggressive nature of the PTC. Histopathologically, there was no pseudocapsule between tumor and surrounding normal thyroid tissues. Therefore, a well-defined tumor margin on delayed contrast-enhanced images usually indicated growth pattern of low aggressive PTC showing a uniform expansion and a relatively intact and thick fibrous stroma around the tumor. Miyakoshi et al^[9]^ have reported that PTC was more often sharply marginated, rather than infiltrative, which was not consistent with our study results. Our study showed that 39 (62.9%) of 62 PTC lesions have poorly or partly defined margin on delayed contrast-enhanced images, and of these, 36 (92.3%) PTCs were highly aggressive. This inconsistency may be due to the differences of observed sequence, MRI protocol and evaluation criteria. Partly or poorly defined tumor margin on delayed contrast-enhanced images was one of the independent factors for the prediction of high aggressive PTC, with a sensitivity, specificity and accuracy of 75%, 78.6%, and 75.8%, respectively.

Tumor size has been established as one of the important factors for tumor risk stratification, with a traditional threshold of 1 cm.^[6]^ We used 1 cm in diameter as a cutoff value for being low or high aggressive PTC, which was based on the range of size of low aggressive PTC. Papillary thyroid microcarcinoma (PTMC) is defined as a tumor of 1 cm or less in size. The disease-specific mortality rates of this have been reported to be <1%, loco-regional recurrence rates of 2% to 6%, and distant recurrence rates of 1% to 2%.^[4,19]^ In the absence of evidence of ETE, metastatic cervical lymph nodes, or distant metastases, PTMC often had an indolent course.^[20,21]^ Hence, as expected, high aggressive PTCs are more likely to be larger than 1 cm in our study (36/48, 75%), while low aggressive PTCs were smaller than 1 cm (12/14, 86%). The mean AUC for tumor size was 0.81, and therefore, tumor size alone is not a very accurate predictor of tumor aggressiveness. In our study, multivariate logistic regression analysis showed that tumor size and margin on delayed contrast-enhanced images were statistically significant variables that are independently used for differentiating high and low aggressive PTCs, with an accuracy of 83.9%.

The movement of water molecules in the biological tissues due to the heat effect is defined as diffusion and Brownian motion. DWI is a noninvasive technique used for measuring the diffusion of water molecules in the tissues.^[22]^ Studies using ADC with DWI for differentiating benign and malignant thyroid nodules are reported in the literature.^[23–26]^ Razek et al^[27]^ in his study demonstrated that sensitivity, specificity, and accuracy of ADC values were used in differentiating benign from malignant nodules (97.5%, 91.7%, and 98.9%, respectively) according to the threshold value. To our knowledge, there was only one article regarding the evaluation of aggressiveness of PTC using DWI. Yonggang Lu et al^[15]^ reported in their study that ADC values of PTCs with ETE were significantly lower than the corresponding values from PTCs without ETE and the cut-off value of ADC to discriminate PTCs with and without ETE was determined at 1.85 × 10^-3^ mm^2^/s with a sensitivity of 85%, specificity of 85%, and AUC of 0.85. These results were in consistent with our study findings. Our study showed that ADC value was significantly different between low and high aggressive PTCs (*P* = .01). However, the mean AUC of ADC was 0.68, which demonstrated that ADC value was not a reliable predictor for differentiating low and high aggressive PTCs. Our study also speculated that different imaging parameters (b-factors) such as equipment, case data and ROI selection led to the differences in conclusion. As far as our experience is concerned, the proportion of different pathological subtypes in the study group might be the main reason for differences.

According to the univariate analysis, angulation on the lateral surface of lesion, signal intensity heterogeneity on ADC maps, enhancement degree on early contrast-enhanced images and the inner lining of delayed ring enhancement were considered to be significant factors, although they were excluded from the independent risk factors in the multivariate analysis. Angulation on the lateral surface of lesion showed a low sensitivity (41.7%) for predicting high aggressive PTC; however, the specificity was up to 100%. Our study showed that all lesions (20) with angulation on the lateral surface showed high aggressive PTC. We speculated that high aggressive tumor tissues showed heterogeneous growth where local growth appeared to be faster as involved in the thyroid capsule, which represented the image findings. In our study, heterogeneous signal intensity on ADC maps was more frequent in high aggressive PTC as compared with low aggressive PTC, which might be a helpful feature of MRI in differentiating high and low aggressive PTCs. Histological patterns when mixed with different pathological subtypes, non-uniform distribution of tumor cells and fiber stroma are more commonly observed in high aggressive PTCs, which are interpreted as heterogeneous signal intensity on ADC maps. Our study showed that all 5 PTCs were hypoenhanced on early contrast-enhanced images and showed high aggressive behavior, while the other 5 PTCs were hyperenhanced and showed low aggressiveness. However, the number of cases was relatively small, and the diagnostic efficacy was uncertain. This might be resolved by increasing the sample number for further evaluation. The inner lining of delayed ring enhancement was significantly related to the tumor aggressiveness, and the indistinct inner lining of delayed ring enhancement was more common in high aggressive PTCs (25/26, 96%), with a sensitivity and specificity of 62.5% and 87.5%, respectively. The fiber stroma around the tumor was more in the central region and usually appeared to be discontinuous. This was interpreted as indistinct inner lining of the delayed ring enhancement, which might in turn help in the prediction of high aggressive PTC.

The study has a few limitations. First, we do not have clinical or follow-up data on the potentially eligible patients who chose not to participate and therefore, an unknown selection bias might exist that influenced our study results. Second, the relatively small number (29%) of low aggressive PTCs limits the statistical power and need further study with a larger number of patients. Third, PTCs with less than 7 mm were not reported in this study, because they are difficult to detect on the MRI images. Improvement in the technique of MRI may help in the detection of smaller PTC lesions. Fourth, most of the features of MRI are qualitative and hence are subjective. Last, there may be differentiating MRI features among different histologic subtypes of PTCs that confound the results of this study, but a subgroup analysis was not possible with the number of patients of specific histologic subtype in this study. In a further study of pre-operative risk stratification of PTC, we will try to complete this part of the investigation.

In conclusion, ADC value, angulation on the lateral surface of lesion, heterogeneous signal intensity on ADC maps, enhancement degree on early contrast-enhanced images and the inner lining of delayed ring enhancement can be used to differentiate high and low aggressive PTCs. Tumor margin on delayed contrast-enhanced images and tumor size are the strongest predictors of aggressive histological features in PTCs. MRI features may provide information for determining PTC aggressiveness.

## Author contributions

**Conceptualization:** Bin Song.

**Data curation:** Bin Song, Hao Wang, Yongqi Chen, Weiyan Liu, Wenjuan Hu.

**Formal analysis:** Bin Song.

**Funding acquisition:** Bin Song.

**Investigation:** Hao Wang, Ran Wei, Zedong Dai, Wenjuan Hu, Yi Ding, Lanyun Wang.

**Methodology:** Bin Song, Hao Wang.

**Project administration:** Bin Song.

**Resources:** Yongqi Chen, Weiyan Liu.

**Software:** Hao Wang.

**Supervision:** Bin Song.

**Validation:** Hao Wang, Ran Wei.

**Visualization:** Hao Wang.

**Writing – original draft:** Bin Song.

**Writing – review & editing:** Hao Wang, Ran Wei.
